# Recurrence of Breast Cancer With Scalp Metastasis

**DOI:** 10.1002/kjm2.70252

**Published:** 2026-06-11

**Authors:** Yi‐Nan Zhu, Wan‐Chen Lu, Zi‐Yue Wang, Xu‐Yong Lin

**Affiliations:** ^1^ Department of Pathology, the First Affiliated Hospital and College of Basic Medical Sciences China Medical University Shenyang China

Dear Editor,

1

Breast cancer is the most prevalent malignancy and a leading cause of cancer‐related death in women worldwide. It may recur locally or metastasize, with common metastatic sites including lymph nodes, liver, and lungs. Cutaneous involvement has also been documented, predominantly presenting on chest skin. Distant cutaneous metastasis of breast cancer remains uncommon. Herein, we report a rare case of isolated scalp metastasis secondary to breast cancer without involvement of other organs.

A 47‐year‐old female presented with a scalp mass that had gradually enlarged over the past 9 months. The lesion was accidentally detected initially with a soybean‐like size and subsequently grew to peanut size. The skin lesion had well‐defined borders, with no apparent surface abnormality compared with adjacent normal skin. The patient received surgical resection for breast cancer 10 years prior. She had no other malignant tumor history, and no relevant familial tumor history was noted. Dermatological examination showed obvious epidermal thickening. Scattered round and oval melanocytes of varying sizes were diffusely distributed with disordered arrangement, accompanied by inflammatory cell infiltration. Histopathological biopsy was recommended to confirm the diagnosis (Figure 1).

Microscopic examination revealed slight keratinization of superficial squamous epithelium, along with abundant diffusely infiltrating tumor cells in the dermis (A). Tumor cells showed enlarged, hyperchromatic nuclei and marked cellular atypia (B).

Immunohistochemical staining showed diffuse positive expression of TRPS‐1 (C), GATA‐3 (D), and CK7 (E). The Ki‐67 proliferation index was approximately 20% (F). ER (G) and PR (H) were strongly positive, with positive rates exceeding 90%. HER‐2 staining was scored 2+ for invasive carcinoma (I). Subsequent systemic imaging examinations of the breast, liver, and lungs detected no additional metastatic lesions. No tumor recurrence or new metastasis was observed during the 6‐month follow‐up period.

**FIGURE 1:**
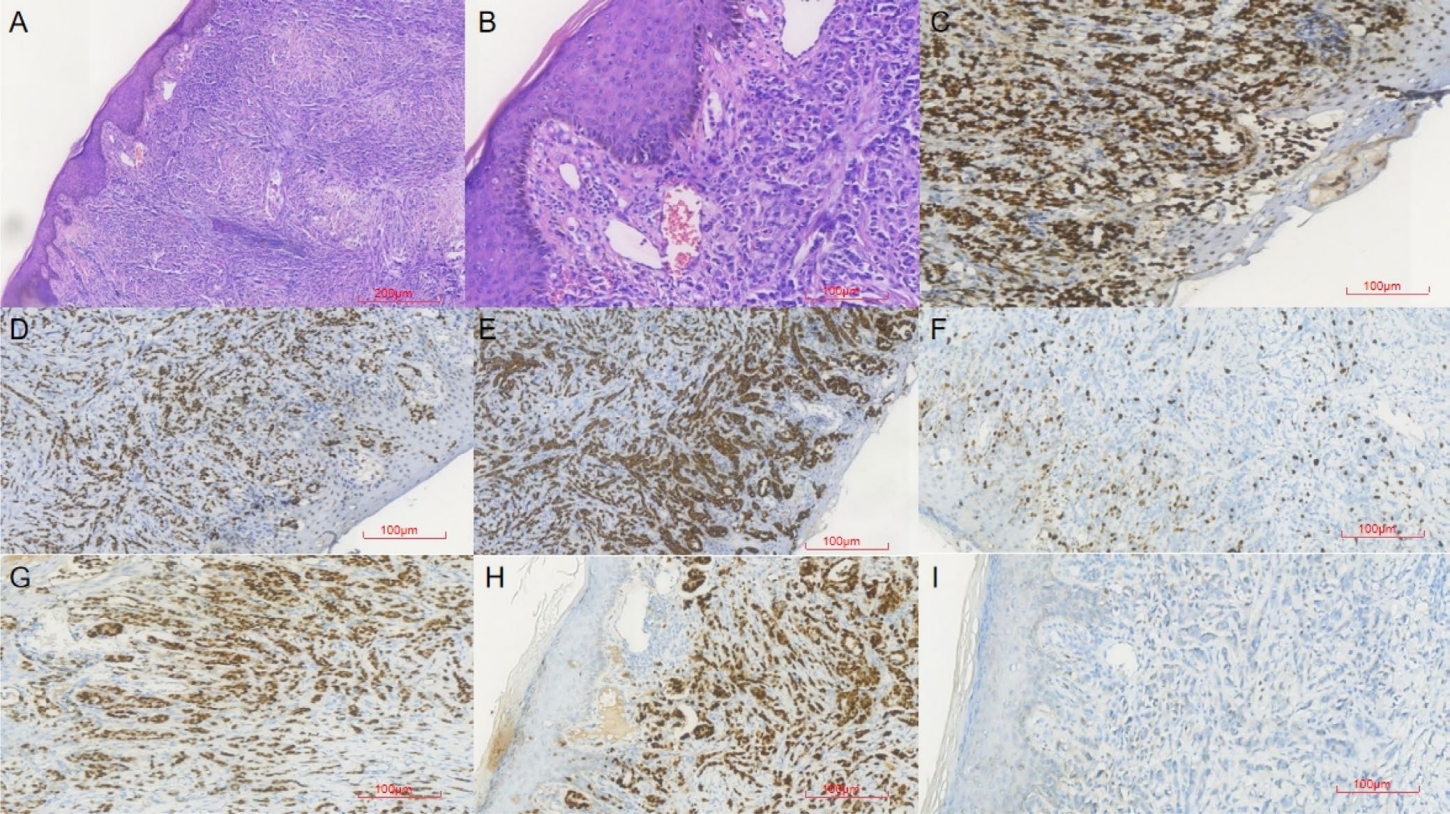
Tumor nests with prominent heterogeneity are observed under the epidermis (A, B). Diffuse positive immunohistochemical staining of TRPS‐1 (C), GATA‐3 (D), and CK7 (E) confirms the breast origin of tumor cells. The Ki‐67 proliferation index is about 20% (F). ER (G) and PR (H) demonstrate strong positive expression. HER‐2 staining result is 2+ for invasive carcinoma (I).

Distant cutaneous metastasis is an uncommon manifestation of breast cancer, as seen in other solid malignancies. Previous case studies indicated that such metastasis usually correlates with unfavorable prognosis and concurrent metastatic lesions in multiple organs [1, 2, 3]. In this case, definite primary breast cancer history was confirmed, while no metastasis was identified in other body sites. Due to non‐specific clinical manifestations, scalp metastatic lesions are easily misdiagnosed as sebaceous cysts, lipomas, basal cell carcinoma, and other cutaneous disorders. Histopathological examination serves as the gold standard for definitive diagnosis. Scalp metastasis is a rare and easily misdiagnosed clinical condition of breast cancer.

## Ethics Statement

The study was conducted in accordance with the ethical standards set forth in the Declaration of Helsinki and was approved by the Institutional Review Board (IRB) of China Medical University. Informed consent was obtained from the patients for the use of their medical data for research and publication.

## Conflicts of Interest

The authors declare no conflicts of interest.

## Data Availability

The data that support the findings of this study are available from the corresponding author upon reasonable request.
